# Metabolic reprogramming: A novel metabolic model for pulmonary hypertension

**DOI:** 10.3389/fcvm.2022.957524

**Published:** 2022-08-26

**Authors:** Xuhan Liu, Liping Zhang, Weihua Zhang

**Affiliations:** Department of Cardiovascular Medicine, The First Hospital of Jilin University, Changchun, China

**Keywords:** PAH, metabolism, mitochondria, randle cycle, glutamine, FAO

## Abstract

Pulmonary arterial hypertension, or PAH, is a condition that is characterized by pulmonary artery pressures above 20 mmHg (at rest). In the treatment of PAH, the pulmonary vascular system is regulated to ensure a diastolic and contraction balance; nevertheless, this treatment does not prevent or reverse pulmonary vascular remodeling and still causes pulmonary hypertension to progress. According to Warburg, the link between metabolism and proliferation in PAH is similar to that of cancer, with a common aerobic glycolytic phenotype. By activating HIF, aerobic glycolysis is enhanced and cell proliferation is triggered. Aside from glutamine metabolism, the Randle cycle is also present in PAH. Enhanced glutamine metabolism replenishes carbon intermediates used by glycolysis and provides energy to over-proliferating and anti-apoptotic pulmonary vascular cells. By activating the Randle cycle, aerobic oxidation is enhanced, ATP is increased, and myocardial injury is reduced. PAH is predisposed by epigenetic dysregulation of DNA methylation, histone acetylation, and microRNA. This article discusses the abnormal metabolism of PAH and how metabolic therapy can be used to combat remodeling.

## Introduction

Idiopathic pulmonary arterial hypertension (IPAH) is a rare type of pulmonary hypertension with a poor clinical prognosis, being classified as group 1 PH (1, 2). The pathologic mechanism of PAH is complex and involves epigenetic factors (3). It is usually dominated by the remodeling of the pulmonary artery or pulmonary vein, or by pulmonary artery combined with pulmonary vein remodeling (4). IPH usually results from pulmonary artery remodeling, whereas pulmonary vein occlusion disease or left heart dysfunction typically results from pulmonary vein remodeling leading to PAH (4). In the vast majority of PAH cases, there is pulmonary vascular remodeling (4, 5). PAH can lead to right ventricular hypertrophy and heart failure, as well as adverse cardiovascular outcomes. In the treatment of pulmonary hypertension, currently available targeted therapeutic agents act on diastolic/contraction balance in the pulmonary arteries. These include SGC stimulants, antagonists of endothelin receptors, and inhibitors of PDE5 (6–8). Despite improving the quality of life in patients with pulmonary hypertension, these drugs did not reverse or delay pulmonary vascular remodeling and still led to the progression of the disease. The cause of pulmonary vascular remodeling can include endothelial cells (EC) dysfunction, cancerous cells proliferating, anti-apoptotic vascular cells, and dysregulated vascular cell metabolism. Researchers initially looked at vascular plexus lesions and the upregulation of HIF-1α in PAH (9) and survivin in IPAH (10). Over the past two decades, the focus of research has shifted from mitochondrial metabolic plasticity to altered intracellular energy metabolism in pulmonary arteries (11, 12). As part of PAH, cellular metabolism is abnormal, including glycolysis, fatty acid metabolism, and glutamine metabolism (13). A metabolic reprogramming mechanism may be involved in PAH pulmonary vascular remodelings. Hence, this review outlines the three abnormal metabolic pathways and identifies possible therapeutic targets for metabolic reprogramming.

## Pathophysiology of PAH

Apoptosis and endothelial dysfunction occur in human PAH patients and animal models, attenuating the endothelial-mediated inhibition of smooth muscle cell proliferation. It has also been found that cloning and amplification of anti-apoptotic endothelial cells can jointly cause angioproliferative lesions (14). Remodeling involves endothelial, mesothelial, and epithelial walls (14) as well as complex interactions between epithelial fibroblasts, perivascular inflammatory cells, and the extracellular matrix. Several other factors have also been described previously as causing pulmonary vascular remodeling, such as genetic mutations, signaling pathway abnormalities, and endothelial dysfunction (6, 15–17). In 1926, Dr Otto Warburg proposed similarities between cancer and PAH. Through *in vitro* and *in situ* observations (18, 19), researchers confirmed the novel cancer-like hypothesis of PAH despite the initial controversy (18, 19). There is evidence that a wide array of growth factors, cytokines, and chemokines contribute to cancer and PAH development. Of these factors, interleukin 6 (IL-6) and platelet-derived growth factor (PDGF) have been extensively studied (20). Within IPAH plexiform lesions, monoclonal amplification of EC and microsatellite sequence instability have been observed (17, 21).

Patients with PAH and cells cultured *in vitro* have chromosomal abnormalities (22). Compared to control cells, smooth muscle cells (SMC) and extracellular vesicles (EC) isolated from PAH patients maintain a longer-lasting anti-apoptotic phenotype (22–24). PVCs from PAH patients exhibit metabolic dysregulation both *in situ* and *in vitro* (11, 12, 25, 26). In summary, the connection between PAH metabolism and proliferation is similar to that found in cancer (14). Moreover, anti-proliferative or anti-cancer agents may play a role in PAH treatment (13). As a result, the therapeutic potential of numerous anti-neoplastic drugs has been tested in preclinical models, with some of them reaching clinical assays (27). The second-generation tyrosine kinase inhibitor dasatinib improves the symptoms of chronic PAH in multiple animal models (27). However, its administration prior to exposure to PH inducers exacerbates remodeling of the pulmonary vascular system and pulmonary artery pressures. Dasatinib alone had no effect on rats' histology or hemodynamics (27).

Under current experimental conditions, more precise data on pulmonary vascular remodeling are still lacking. As a molecular marker for veins, hepatic ligand protein B4 receptors are currently ineffective at accurately determining pulmonary veins (4). Therefore, it is necessary to develop more effective experimental tools for assessing pulmonary vascular remodeling. Furthermore, many of the vascular cells of PAH in established humans are quiescent and may resist apoptosis. Quiescent cells have different metabolic requirements than aberrantly proliferating cells (4). The field must therefore clarify the cause of metabolic heterogeneity and how it contributes to vascular remodeling in the future (4).

## PAH metabolism

The metabolism of PAHs is accomplished by many cellular and molecular mechanisms with mitochondria at their cores. Metabolic reprogramming is an emerging hallmark of oncogenesis (28). Growing evidence suggests that metabolic dysfunction may be associated with changes in PAH expression and susceptibility (4). Researchers examined metabolite changes in hypoxia-induced PASMCs and PDGF-BB-induced PASMCs (29, 30). A recent report on metabolic alteration was also explored in perioperative period of congenital heart disease associated with pulmonary arterial hypertension (CHD-PAH) patients undergoing repair (31). Furthermore, patients with PAH may be more sensitive to mutagens because of impaired DNA-response mechanisms (32). There was a correlation between PAH and decreased expression of breast cancer 1 protein (BRCA1) and DNA topoisomerase 2-binding protein 1 (TopBP1), both of which are involved in the integrity of the genome (32). Dedicated to overcoming the antiproliferative and lethal effects of DNA damage, PAH-PASMCs have developed a network of highly efficient and complementary mechanisms (33), including sanitation of the oxidized nucleotide pool (the result of this study), DNA damage sensing, and repair (33). Metabolomics studies also provide a detailed understanding of metabolic disturbances in experimental PH challenged by MCT administration or hypoxia (34, 35).

Prior to PAH, cellular cancer-like metabolic abnormalities were thought to include aerobic glycolysis, pentose phosphate pathway activation, mitochondrial dysfunction, altered fatty acid metabolism, and insulin resistance. In PAH, mtHSP90 accumulation contributes to vascular remodeling through its regulation of mitochondrial homeostasis (36). With further research in the area of metabolic dysregulation in PAH, Warburg's principle of dysregulated metabolism can now be included in the metabolic theory of PAH as well as other dysregulated pathways. Earlier studies hypothesized that metabolic dysregulation would occur in the pulmonary vasculature. Even so, increasing evidence suggests that RV metabolism may be present in muscle tissue, which indicates that PAH development may be the result of a combination of paracrine and systemic actions. To develop a comprehensive metabolic theory of PAH, it is necessary to discover the nature of metabolic disorders outside the pulmonary vascular system. PAH has been associated with metabolic dysfunction since its inception, according to many early studies. PAH pathology is characterized not only by hyperproliferative and apoptosis-resistant PVCs, ECs, and SMCs, but also by other types of cells in the vascular system. Cancer-like proliferative increases and resistance to apoptosis are indicative of abnormal mitochondrial metabolism and dynamics (37). A dysregulation of mitochondrial metabolism occurs in the early or late stages of PAH, and its link to reprogramming remains unclear.

Metabolism and inflammation: accumulating evidence suggests a functional role of perivascular inflammation in the initiation and/or progression of pulmonary vascular remodeling (38, 39). There is a correlation between clinical outcomes and high levels of cytokines, chemokines, and inflammatory mediators in PAH patients (38). In PAH lung biopsies, macrophages, mast cells, and T lymphocytes were detected near remodeled pulmonary vasculature, mainly in proximity to macrophages, mast cells, and T lymphocytes (40, 41). Consequentially, several clinical trials have been conducted to investigate inflammation and autoimmunity treatments in PAH patients (42). However, the exact role of inflammation and immunity in PAH-and specifically, its function as a cause, promoter, or downstream bystander—is poorly understood and remains a contentious issue.

RVH is commonly out of adjustment, with two exceptions: ischemia and aerobic glycolysis (43). The Warburg effect is associated with molecular events that adapt to acute cellular stress and prevent apoptosis. In addition to metabolic dysregulation, recent studies have revealed metabolic abnormalities in maladaptive RVH, including aerobic glycolysis, glutamine metabolism, and the Randle cycle (44). Pyruvate kinase muscle isozyme 2 (PKM2), already known for its role in pulmonary artery obliteration, was identified as an integrator, of anaerobic metabolism, oxidative stress, inflammation, and fibrosis in several diseases sharing with RV failure (45). The energy metabolism in PAH needs to be capable of meeting increased metabolic demands, which requires enhanced aerobic glycolysis and reprogramming of multiple molecules. PAH plasticity and adaptation should be combined with the control of cellular stress and protein misfolding, and protein sorting in intracytoplasmic compartments (4), with implications for treating PAH. Below we will describe three types of abnormal metabolism and their relationship with metabolic reprogramming.

## Aerobic glycolysis

In PAH, altered intracellular glucose transport may contribute to glucose intolerance. The presence of glycated hemoglobin (46, 47) in IPAH suggests that glucose intracellular influx and insulin resistance are always present when chronic hyperglycemia is present (15, 20, 34, 35). Studies have shown that cancer and PAH share similar features (18, 48–50), including increased cellular glucose uptake and altered glucose metabolism (51). Proliferating cancer cells and pulmonary vascular endothelial cells use a great deal of energy. Under normal oxygen partial pressure, highly proliferating cells change the metabolism from glycolysis to aerobic glycolysis, or the Warburg effect (52). When glucose is available, aerobic glycolysis increases the survival rate of proliferating cells and enhances glucose metabolism. The results of (18F)-fluorodeoxyglucose-PET (FDG-PET) showed that IPAH patients had higher pulmonary glucose uptake and a higher glycolysis ratio than normal subjects (53). Compared to normal cells, endothelial cells had a 3 times higher glycolysis rate, which might improve the proliferation rate of abnormal cells (25). It can therefore be believed that PAH has abnormal aerobic glycolysis, which can result in enhanced glucose utilization, which is used to meet the proliferating cells' energy requirements.

In PAH patients, glucose is mainly metabolized by aerobic glycolysis. In pulmonary vascular cells of PAH animal models and IPAH patients (54), Diebold et al. found hyperpolarized mitochondria, which resulted in diminished oxidative phosphorylation and glucose oxidation. A metabolic pathway that is abnormal in PAH patients, aerobic glycolysis is likely caused by mitochondrial dysfunction (55). In PAH patients and in preclinical PAH models, positron emission tomography has shown increased glucose uptake in the lungs and right ventricle by Warburg metabolism (53). Glucose influx may be caused by increased glucose transporter protein (Glut) expression (56, 57) and expression of a splice variant of terminal pyruvate dehydrogenase kinase (PDK) (57), act together to upregulate the glycolytic response in PAH (56, 57). Glycolysis produces pyruvate. Pyruvate dehydrogenase (PDH) is a key enzyme involved in the complete oxidation of pyruvate to coenzyme A. Inhibits the flow of pyruvate into mitochondria and the oxidation of glucose inside mitochondria. Inhibition of PDK function occurs through phosphorylation of E1-α by PDK (58). Increased expression of PDK inhibits acetyl coenzyme A formation and the Krebs cycle and oxidative phosphorylation in mitochondria in PAH (55). PDK-mediated metabolism impairs cardiac mechanical performance and electrical remodeling, as a result, right heart contractility is reduced, cardiac output is reduced, and RV action potential and QT intervals are prolonged (59). DCA inhibits PDH function by reducing PDH phosphorylation (59), which improves glucose oxidation, RV contractility, and PAH vascular remodeling. Theoretically speaking, DCA corrects pathological inhibition of PDH and glucose oxidation responses only in the lungs and renal vasculature (60), and has little effect on the normal heart and vasculature. Accordingly, mitochondrial hyperpolarization leads to aerobic glycolysis in patients with PAH. Glut expression and PDK overexpression cause an increase in glucose in-flow. DCA targets the lung and RV, affecting energy supply, decreasing RV function, and aggravating ischemia (54, 59). Thus, active control is necessary.

Associated with PAH metabolism disorders caused by a variety of changes in the structure and content of glucose derivatives. Patients with COPD had altered structures of glycoprotein glycans (61). O-GlcNAc level and function are altered in IPAH as a result of altered glucose uptake (62). The hypoxia-induced enhancement of glycosaminoglycan (GAG) synthesis in human lung fibroblasts may contribute to the regulation of GAG synthesis/deposition in PAH. UDP-GlcNAc regulates the synthesis of HA, a large GAG (63). Plasma HA levels are elevated in patients with IPAH (64) and may be related to vascular remodeling in PAH and inflammatory response processes (65). The HSPGs perlecan and agrin are elevated in PAH patients (66). The perlecan content of the EC-PASMC junction is higher (67), and it may be involved in EC barrier function in PAHs, EC-PASMC interactions, and cell proliferation inhibition (67). There may be an association between changes in the structure and content of various glucose derivatives in PAH patients and inflammation and vascular remodeling, as well as vascular plexiform lesions. Despite the late start to the study of the above substances in PAH, more research is needed to determine their specific roles and therapeutic targets. According to Warburg in 1926, aerobic glycolysis, a metabolic change in the pulmonary vascular system, is regulated by the redox state of the activating transcription factors. In addition to HIF-1α, episodic silencing of superoxide dismutase 2 (SOD2) and mitochondrial fission/fusion are also associated with PAH. The oxidative metabolism is inhibited and sustained glycolysis is favored (68).

### HIF

Oxygen-sensitive prolyl hydroxylase structural domain-containing enzymes (PHDs) regulate HIF subunit expression (69), which are hydroxylated under aerobic conditions. Von Hipple-Lindau protein (VHL) ubiquitinates targeted HIF subunits (69).

HIF-1α activation may result from abnormalities in mitochondrial metabolism in PAH (70). The abnormalities reduce hydrogen peroxide production and eliminate inhibition of HIF-1α activation (71). DNA methyltransferase in the lung activates epigenetic silencing of superoxide dismutase (SOD2) (71), interfering with gene transcription and reducing hydrogen peroxide levels in the blood, activating HIF-1α and initiating glycolysis. Under normoxia, inhibition of SOD2 expression with siRNA activated HIF-1α in normal PASMC (71). By applying 5-azacytidine for demethylation, SOD2 expression was restored and PASMC proliferation was inhibited (72). A dysregulation of DNA methyltransferase was observed in PAH assays, which may be tissue specific.

**Figure 1:**
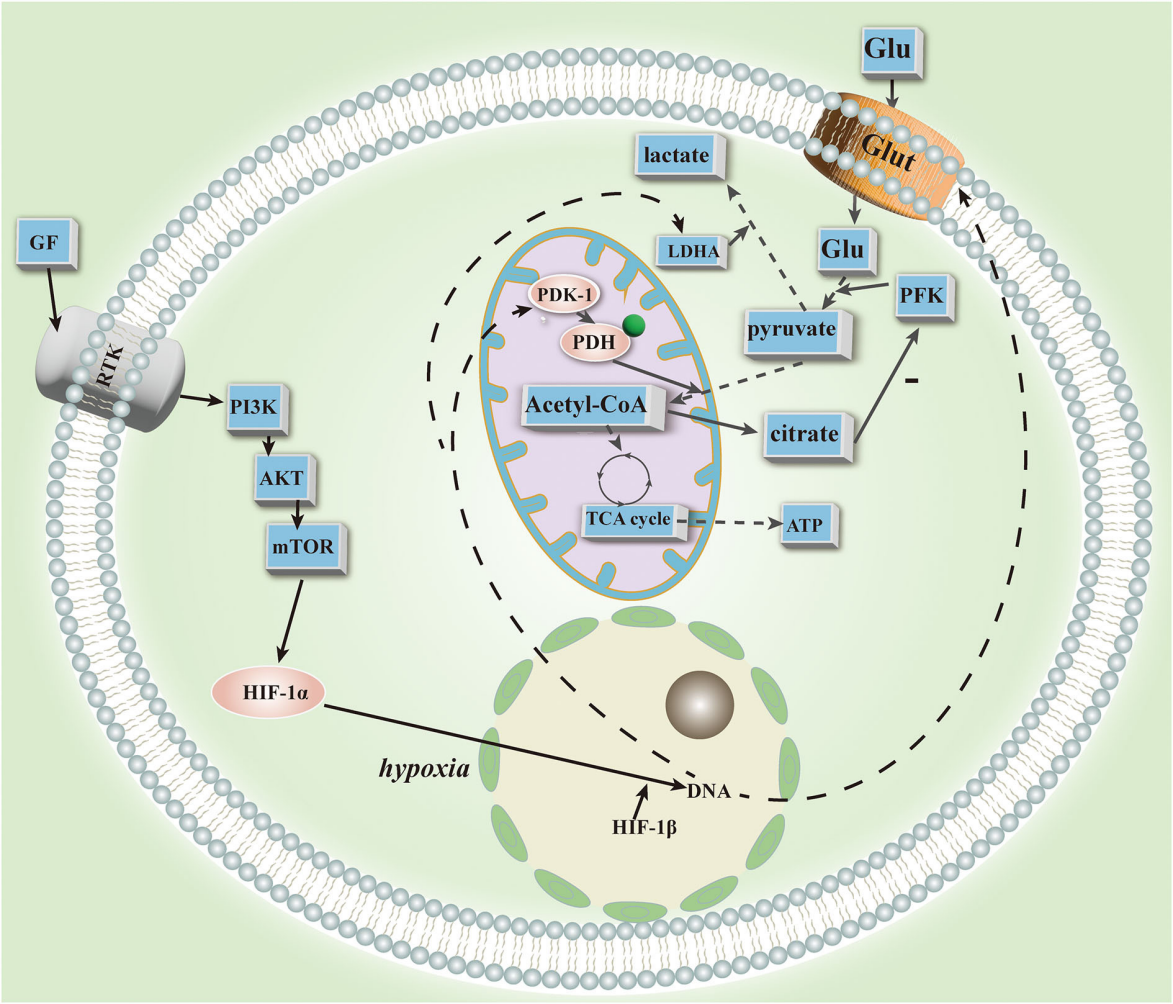
This figure depicts the major steps and key substrates in aerobic glycolysis.

In PAH, HIF-1α is closely related to mitochondria. HIF-1α activation with cobalt or desferrioxamine results in mitochondrial fission in human and rodent PAH smooth muscle cells (73), suggesting its association with mitochondrial plasticity. In addition, it regulates mitochondrial dynamics, which results in a decrease in mitochondria and reduced NO utilization in IPAH cells (9). The HIF promotes further lung endothelial damage by mobilizing hematopoietic precursors in PAH. In the mitochondrial respiratory chain, HIF-1α promotes the expression of cytochrome oxidase subunit 4.2 (74). Furthermore, it upregulates PDK transcription (75) and inhibits PDH function to produce a glycolytic state (76). The source of HIF-1α stability is unknown, but it has been demonstrated to be inherited (70). Studies have recently confirmed the significance of HIF-2 in PAH animals. HIF-2 is upregulated and BMPR2 and Cav1 are downregulated in PHD-2 deficient EC under normoxia (77). HIF-2 facilitates the upregulation of the chemokine CXCL12, which stimulates PASMC proliferation (77). HIF-2 deletion also reverses PAH (77). Deletion of HIF-2 is associated with reduced expression of arginase (Arg1), and Arg1 deletion also reverses PAH (72). Transcriptional factor POU5F1 (or OCT4) targets HIF-2 (78), and hypoxia and inflammatory factors (such as IL-1b and IL-6) can upregulate miR-130/301 in EC and PASMC, respectively. Rodent models and patients' lungs and plasma have confirmed this (78). An analysis of bioinformatics data revealed that members of the miR-130/301 family regulate other miRNAs and cellular phenotypes in PAH. *In vitro* expression of miR-130a in human EC and PASMC has demonstrated this effect. MiR-130/301 inhibits peroxisome proliferator-activated receptor-γ (PPARγ). PPARγ is a target of BMP signaling (79), and inhibition causes abnormal proliferation of vascular endothelium. It may also regulate STAT3-miR-204-SRC pathways (80) and apelin-miR-424/503-FGF2 pathways, maintaining EC homeostasis and inhibiting vascular endothelial proliferation (81). In the glycolytic state, HIF-1 and HIF-2 act together through different mechanisms in the pulmonary vascular system. As a result of HIF-2, miRNAs are important players in glycolysis, and several miRNA inhibitors or mimics have shown therapeutic efficacy in animal models. Despite their lack of tissue specificity, inability to penetrate and degrade cells, and potential toxicity, much preliminary work is still needed before they can be used for deep tissue regeneration (Figure 1).

## Glutamine metabolism

Research examining mitochondrial dysfunction and aerobic glycolysis partially explains the aberrant energy metabolism in PAH. YAP (Yes-associated protein 1) was recently found to be correlated with glutamine metabolism and vascular proliferation. Yes-associated protein 1 (YAP), when containing a PDZ-binding gene sequence (TAZ), stimulates pulmonary vascular smooth muscle and endothelial proliferation (82). There was increased expression of GLS1 in small pulmonary arteries of rats treated with wild lily base (MCT)-induced PAH and decreased glutamine in isolated EC in this model (82). An additional study found increased glutamine catabolism in patients with PAH and in rats implanted with MCT-induced RV (83). Krebs cycle increases glutamine catabolism while also reshaping glutamate metabolism, possibly contributing to the loss of compensatory cardiac remodeling response. Both monkey HIV-associated PAH and human HIV-mediated PAH were associated with elevated GLS1 expression in the lungs of rhesus monkeys (84). In addition, glutamine catabolism results in proline hydroxylation and α-ketoglutarate activation of mammalian target of rapamycin (mTOR) proteins to promote collagen stability and translation and enhance fibrosis (85), leading to vascular hyperproliferation. Contrary to this, pharmacological inhibition of GLS1 activity affects the Krebs cycle and reduces MCT-induced arterial remodeling in rat PAH (83). GLS1 was inhibited and PAH improved in clinical trials with anticancer drugs (NCT02071862) (86), MK-801 (an NMDAR) (81), and vetiprofen (a YAP) (82). A treatment option that could be considered is reprogramming glutamine metabolism.

There is a significant association between mutations in the bone morphogenetic protein receptor type II gene (BMPR2) and hereditary PAH (37). It has been suggested that aberrant bone morphogenetic protein signaling and epistasis promote aerobic glycolysis for cell proliferation (37). Egnatchik et al. found that PAH patients with aberrant BMPR2 gene mutations had lower trans-lung glutamine concentrations.

BMPR2 gene mutations may affect glutamine metabolism based on the large reduction in glutamine. Yelamanchi et al. suggested that EC with BMPR2 mutations fails to tolerate glutamine-limited survival conditions (87). They suggested that glutamine dependence is caused by mitochondrial damage. Mutations of BMPR2 drive downstream and lead to the formation of ecdysone (87). When Ecdysone is removed from the body, glutamine metabolism returns to normal. It stabilizes HIF-1 and inhibits Sirtuin-3.

Cancer-like glycolysis results when PAH metabolism is dysregulated, causing a shift from oxidative phosphorylation (OXPHOS) to aerobic glycolysis (Warburg effect), and glutamine metabolism, and increased fatty acid oxidation in mitochondria. Based on earlier studies, pulmonary vasculature could provide sufficient glucose for aerobic glycolysis, which would supply energy for cell proliferation. But as glycolysis is enhanced in the Krebs cycle, there is a decrease in the supply of carbon substrates for nucleotides and proteins, and a concomitant decrease in the carbon intermediates not involved in ATP formation, which is not consistent with increased vascular cell proliferation. Thus, it has been hypothesized that glutamine catabolism is essential for backfilling the Krebs cycle with carbon intermediates. Catabolizing glutamine provides carbon intermediates for the Krebs cycle, which provides lipids and amino acids for nucleotide biosynthesis (88). The upregulation and uptake of glutamine by GLS1 drive PAH. PAH is then utilized for glutamate to produce ^*^-ketoglutarate for the Krebs cycle. On the other hand, YAP and TAZ are essential for glutaminase upregulation and hydrolysis. The activation of YAP/TAZ leads to metabolic reprogramming of PAECs and PASMCs *in vitro*, enhancing glycolysis and glutamine catabolism. This process plays an important role in maintaining a proliferating cell phenotype (78).

The glutamate receptors (GluR) are responsible for the function of glutamate, a non-essential amino acid (89). NMDAR and mGluR5 have been found to be located in the pulmonary vasculature and RVs, suggesting they may play a role in the pathogenesis of PAH. The ionotropic glutamate receptor (iGluR) is a glutamate-activated ion channel. iGluR-associated membrane channels, including Na+, K+, and Ca^2+^ channels, exhibit certain physiological and pharmacological characteristics. By causing Ca^2+^ ion inward flow and activating protein kinase C and its AKT/MAPK pathway, the NMDA receptor (NMDAR), a type of pulmonary vascular iGluR, causes PAH remodeling. Proliferation and anti-apoptosis are the results. By inhibiting NMDAR, the progression of PAH could be stopped and vascular remodeling reversed. The metabolic glutamate receptors (mGluRs) are another type of glutamate receptor, classified into four groups. The mGluRs are attached to intramembrane G proteins and act on second messenger synthesis or ion channels by activating GTP-binding proteins. Group I mGluR stimulates inositol triphosphate (IP3) metabolism and Ca2+ transfer (90). This signaling pathway activates IP3 and stimulates the intracellular release of Ca2+. mGluR5 is involved in this pathway. Adenylate cyclase (AC) is associated with Group II and III mGluR. Vasodilation is promoted by prostacyclin (PGI2). AC activation increases the level of cAMP, which activates protein kinase A (PKA), which restores the activity of the phosphorylation pathway, and reduces the metabolic shift toward glycolysis. Accordingly, it appears that GluR plays an important role in the pathogenesis of PAH and may therefore affect its prognosis by regulating GluR.

There are targeted therapies for tumors that inhibit glutamine metabolism, such as the GLS inhibitors BPTES and CB-829 (in phase I clinical trials: NCT02071862 and NCT02771626), that suppress tumors in preclinical animal models (91). Studies in preclinical and clinical settings have shown that glutamate dehydrogenase inhibitors, transaminase inhibitors, and glutamine analogs reduce the production of α-ketoglutarate and nucleotides in the Krebs cycle (92). 2-Aminobicyclo(2,2,1)-heptane-2-carboxylic acid (BCH), an inhibitor of the glutamine transporter protein SLC7A, is also a great therapeutic option. By inhibiting the mTOR signaling pathway, BCH prevents glutamine-induced fibrosis from forming (93). As a result, it blocks activation of the mTOR pathway by ^*^-ketoglutarate and inhibits proline hydroxylation by PAH (85). 000In conclusion, glutamine metabolism replenishes carbon intermediates used in aerobic glycolysis and promotes PAH progression. Clinical trials and *in vitro* studies have investigated glutamine metabolism's antitumor effects. We should examine in more depth in the future the reprogramming of glutamine metabolism, the inhibition of glutaminolytic enzymes, and the glutamate receptors as therapeutic targets.

## Randle cycle

Throughout the adult heart, fatty acid oxidation (FAO) supplies ATP. The Randle cycle (94) occurs when fat in the blood inhibits cellular glucose metabolism. FAO produces citrate, which inhibits phosphofructokinase, increasing glucose-6-phosphate levels, inhibiting hexokinase, and reducing pyruvate production. In addition, FAO production of acetyl coenzyme A inhibits PDH. Stephen L. et al. measured direct myocyte metabolism and discovered that FAO and GO inhibition led to a metabolic shift to glycolysis *in vivo* (68). Two FAO inhibitors, trimetazidine, and ranolazine, did not prolong the QTc interval in a rat model of PAH with ligated pulmonary arteries and increased GO and raised ATP levels, suggesting that FAO is detrimental (95). FAO inhibition activates the Randle cycle (95). Hou posited that FAS inhibition protects mice from PAH caused by hypoxia through activation of P13/AKT signaling through C73 (96). The amount of glycogen in the RV increases when GO is inhibited, and it decreases when FAO is inhibited (95). Guarnieri suggested that trimetazidine has a positive effect on the MCT-induced PAH model (96). The absence of malonyl coenzyme A decarboxylase (MCD) inhibits FAO oxidation and promotes glycolysis, preventing the “glycolytic shift” that occurs during hypoxia. Trimetazidine is metabolized similarly to PAH in rats treated with trimetazidine (26). Inhibiting FAO metabolism, increasing GO metabolism, and activating the Randle cycle will therefore benefit PAH treatment. The use of trimetazidine and ranolazine for the treatment of PAH patients, as well as the combination of FAO inhibitors with PDK inhibitors, requires further study.

## Conclusion and prospect

Previously, PAH studies focused on the NO-TAX2-ET1 pathway, but in recent years, metabolic dysregulation and reprogramming have gradually gained attention. Spermine synthase inhibition inhibited platelet-derived growth factor-BB-mediated PASMC proliferation *in vitro* and reduced monocrotaline-induced pulmonary hypertension in rats *in vivo*. A therapy for PAH that inhibits spermine synthesis would promote pulmonary vascular remodeling (97). Glut overexpression resulted in increased glucose in-flow and PDK overexpression resulted in mitochondrial hyperpolarization related to aerobic glycolysis. A large amount of carbon intermediates is consumed during aerobic glycolysis, which does not produce enough energy. Energy and carbon intermediates are replenished by glutamine metabolism, which are supplied to proliferating cells and anti-apoptotic cells. In cardiac myocytes, FAO is responsible for the majority of energy, and inhibiting FAO will enhance GO and prevent PAH. The major source of energy in cardiac myocytes is FAO, and inhibiting FAO will increase GO and prevent hypoxia that leads to PAH. Consequently, therapeutic agents targeting the three metabolic pathways and metabolic reprogramming hold great promise in PAH. The use of certain drugs for the treatment of diabetes and metabolic disorders may also be appropriate for PAH. It is still necessary to study their safety and potential side effects further.

## Author contributions

XL: methodology, investigation, formal analysis, and writing-original draft. LZ: writing-review and editing. WZ: project administration and supervision. All authors contributed to the article and approved the submitted version.

## Conflict of interest

The authors declare that the research was conducted in the absence of any commercial or financial relationships that could be construed as a potential conflict of interest.

## Publisher's note

All claims expressed in this article are solely those of the authors and do not necessarily represent those of their affiliated organizations, or those of the publisher, the editors and the reviewers. Any product that may be evaluated in this article, or claim that may be made by its manufacturer, is not guaranteed or endorsed by the publisher.
